# Status of Workers’ Health Behavior and the Association between Occupational Characteristics and Health Behavior

**DOI:** 10.3390/ijerph192013021

**Published:** 2022-10-11

**Authors:** Seung-Yeon Lee, Saemi Jung, Wanhyung Lee

**Affiliations:** 1 Department of Family Medicine, International Healthcare Center, Seoul National University Bundang Hospital, Bundang-gu, Seongnam 13620, Korea; 2 Department of Occupational and Environmental Medicine, Pusan National University Yangsan Hospital, Yangsan 50612, Korea; 3 Department of Occupational and Environmental Medicine, Gil Medical Center, Gachon University College of Medicine, Incheon 21565, Korea

**Keywords:** occupational health, lifestyle, smoking, alcohol drinking, exercise, physical activity, KNHANES

## Abstract

This study investigated differences in unhealthy lifestyle behaviors (ULBs) between workers and nonworkers and demonstrated the association of ULBs with occupational characteristics among workers. This cross-sectional study used data from the Korea National Health and Nutrition Examination Survey from 2007 to 2019. For sociodemographic data, chi-squared tests were used to analyze categorical variables. Odds ratios (ORs) and 95% confidence intervals (CIs) for ULBs were estimated using Poisson regression models after adjusting for age, sex, educational level, and household income. The variables used were current smoking status, heavy drinking, and physical inactivity. Workers were associated with an increased risk of current smoking (adjusted OR (aOR) = 1.48, 95%CI = 1.41–1.56), heavy drinking (aOR = 1.79, 95%CI = 1.68–1.90), and physical inactivity (aOR = 1.07, 95%CI = 1.04–1.11) compared with nonworkers. Among workers, the differential risks of ULB according to occupational characteristics were as follows: skilled manual workers, self-employed workers, and workers working >40 h/week were at a higher risk of engaging in all ULBs than those in other occupational categories, paid workers, and workers working ≤40 h/week, respectively. Workers showed a higher risk of ULBs than nonworkers. The risk of ULBs differed according to occupational characteristics, highlighting the need for additional studies and detailed occupational health management.

## 1. Introduction

Individual behavior has a strong influence on a person’s risk of developing a range of diseases [1,2]. Physical activity (PA), alcohol consumption, and cigarette smoking are among the most important behavioral determinants of health [3,4]. Several studies investigating the independent and combined health effects of lifestyle behaviors have demonstrated that healthy behaviors are associated with a lower incidence of chronic diseases, reduced premature mortality, and prolonged life expectancy, while the opposite is true for risky behaviors [5,6]. According to a large-scale longitudinal study, participants who engaged in four healthy lifestyle behaviors (smoking status, body mass index, PA, and diet) had a 78% lower risk of developing chronic diseases than those who did not— diabetes (93%), myocardial infarction (81%), stroke (50%), and cancer (36%) [7]. A systematic review and meta-analysis of 15 studies including 531,804 participants from 17 countries showed that the combination of at least four healthy lifestyle behaviors was associated with a 66% reduction in the risk of all-cause mortality [8]. Parry et al. suggested that alcohol consumption is responsible for 3.4% of noncommunicable disease-related deaths [9]. Beaglehole et al. reported that smoking accounts for a sixth of noncommunicable disease-related deaths [10]. Lee et al. estimated that physical inactivity causes 6% of coronary heart disease, 7% of type 2 diabetes, 10% of breast cancer, 10% of colon cancer, and 9% of premature deaths worldwide [11]. 

Lifestyle behaviors are associated with a variety of factors, including sex, personality, socioeconomic status, and workplace environment [12]. The workplace is an important setting because most adults spend most of their waking hours at work. Many studies have attempted to estimate the influence of work on health, and some have suggested that individual health behaviors are affected by working conditions [13]. Stressful working conditions can promote unhealthy lifestyle behaviors (ULBs), including smoking, heavy drinking, and physical inactivity [14,15]. A recent systematic review found that the weakness of safety behavior was associated with accidents and injuries, and that safety climate can improve safety behavior [16]. Safety behaviors and awareness among workers are important factors in promoting workplace safety and health [17,18]. 

Although numerous studies have investigated the relationship between workplace stress and ULBs, few have compared the prevalence or risk of ULBs between workers and nonworkers, with further categorization based on occupational characteristics, such as occupational classification, employment status, working schedule, and weekly working hours. Therefore, we designed this study to investigate the differences in ULBs between workers and nonworkers and further demonstrate the associations of ULBs with occupational characteristics using Korean national representative data.

## 2. Materials and Methods

### 2.1. Data and Study Participants

**Figure 1:**
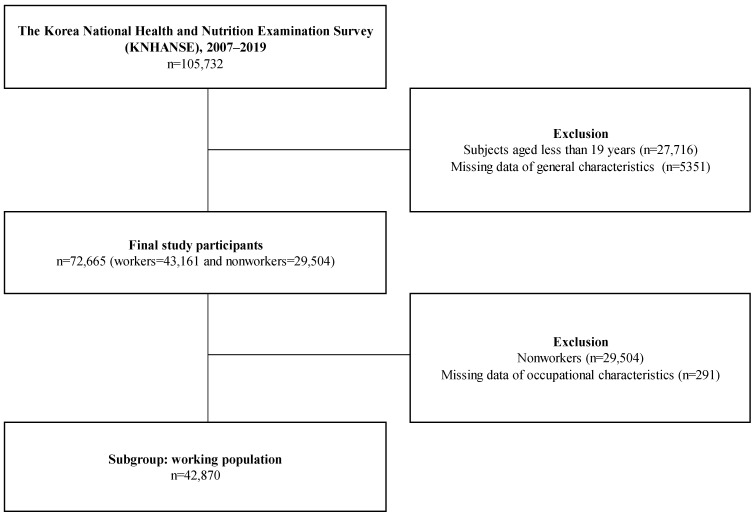
Schematic diagram depicting the selection of the study population.

This study was performed using data from the Korea National Health and Nutrition Examination Survey (KNHANES), which evaluates approximately 8000–10,000 individuals from approximately 4000 households each year to characterize the health and nutritional status of Koreans. The KNHANES is managed by the Korea Centers for Disease Control and Prevention, and the data are publicly available after anonymization to prevent individual backtracking or leakage of personal information [19]. This study used data from 105,732 KNHANES participants between 2007 and 2019. After excluding 27,716 individuals aged less than 19 years and 5351 individuals who had missing data or refused to provide data for the study items, the data for the remaining 72,665 participants were used in the analyses. To demonstrate detailed occupational characteristics related to ULBs, we used the data of 42,870 workers from the study population in subgroup analyses. Figure 1 shows a schematic diagram of the study population.

### 2.2. ULBs 

We evaluated three ULBs: smoking, alcohol consumption, and PA. Smoking was categorized as current smoking or not, and current smokers were defined as those who reported smoking cigarettes at the time of the survey. Alcohol consumption was categorized as heavy drinking or not, and heavy drinking was defined as alcohol consumption over 30 g/day [20]. PA was categorized as active or inactive; inactivity was defined as activity levels under the threshold for moderate physical activity in the Korean version of the International Physical Activity Questionnaire, i.e., five or more days of weekly moderate-intensity activity of at least 30 min per day [21]. 

### 2.3. Occupational Characteristics 

The current analysis evaluated four occupational characteristics: occupational classification, employment status, work schedule, and weekly working hours. The International Standard Classification of Occupations proposed by the International Labour Organization in 2008 categorizes occupations into 10 major groups based on the nature of the task and job duties. This study used a modified occupational classification with six categories, based on a previous study [22]. The manager group included legislators, senior officials, managers, and professionals. The office worker group comprised technicians, associate professionals, and clerical support workers. Service and sales workers included sales and service professionals. Agricultural and fishery groups included skilled agricultural, forestry, and fishery workers. The skilled manual worker group consisted of craft and related trade workers, plant and machine operators, and assemblers, whereas the simple manual worker group included workers engaged in elementary occupations. Those with armed forces occupations were excluded from this study. Employment status was classified into paid workers, self-employed, and others, according to the type of remuneration. The working schedule was defined using questionnaire responses regarding working time schedule and categorized as daytime fixed and shift schedules. Weekly working hours, excluding meal or break times, were surveyed in the KNHANES and categorized as less than 40, 41–60, and over 60 h. 

### 2.4. Covariates 

This study used four sociodemographic characteristics (e.g., age, sex, educational level, and income level) as covariates. Educational level was categorized as middle school graduate or lower, high school graduate, and college or higher. Income level was categorized based on the household income status quartiles. 

### 2.5. Statistical Analysis

The prevalence of each ULB in relation to sociodemographic status and occupational characteristics was calculated using the chi-squared test. A Poisson regression model was used to analyze the association between working status and each ULB. Adjusting factors were selected by best model fitting with backward step wising. All results from the regression analysis were conducted after adjusting which were age, sex, educational level, and household income as confounders in the regression model. The fully adjusted Poisson regression model for the association between occupational characteristics and each ULB was used for subgroup analysis with only workers. All analyses were performed using SAS, version 9.4 (SAS Institute, Cary, NC, USA). For all statistical calculations, a two-tailed *p*-value < 0.05 was considered statistically significant. 

## 3. Results

Table 1 depicts the prevalence of ULBs according to sociodemographic characteristics of the study participants (n = 72,665). The overall prevalence rates of current smoking, heavy drinking, and physical inactivity were 19.4% (n = 14,117), 10.9% (n = 7961), and 59.8% (n = 43,482), respectively. All study variables (sex, age, education level, household income, and working status) showed statistically significant differences (*p* < 0.05) in the distribution of each ULB. Current smoking and heavy drinking were more prevalent in males, younger individuals (19–40 years), and those with education up to high school. PA was more prevalent in females, the middle-age group (41–60 years), and those with education less than middle school. The prevalence of current smoking and heavy drinking was higher in the third and fourth quartiles of household income, respectively, while physical inactivity was more common in the lowest income group. Workers were more often current smokers and heavy drinkers than nonworkers. However, physical inactivity was more prevalent among nonworkers.

Additional descriptive data analyses were conducted separately for each working population. The prevalence of ULBs according to sociodemographic and occupational characteristics among the workers is shown in Table 2. Of the 72,665 study participants, 42,870 (59.4%) were employed. The overall prevalence of current smoking, heavy drinking, and physical inactivity among the workers was 24.7% (n = 10,570), 14.6% (n = 6238), and 59.7% (n = 25,596), respectively. Males showed a higher prevalence of current smoking and heavy drinking than females. There were no significant sex-related differences between the physically active and inactive individuals. Workers in the younger age group (19–40 years) and the middle-education group (up to high school) showed a higher prevalence of current smoking and heavy drinking. Physical inactivity was more prevalent in the middle-aged group (41–60 years), but its prevalence did not differ significantly according to education level. The prevalence of current smoking, heavy drinking, and physical inactivity was higher in the third and fourth quartiles of household income. Skilled manual workers showed a higher prevalence of current smoking and heavy drinking (*p* < 0.05) than workers in the other occupational categories. However, the prevalence of physical inactivity did not significantly differ across occupational categories. Regarding employment status, self-employed workers showed a higher prevalence of current smoking, heavy drinking, and physical inactivity than did paid workers. Shift workers were more often current smokers and heavy drinkers than day workers, whereas day workers were more often physically inactive than shift workers. All ULBs were more prevalent in workers working over 60 h/week than in those working 41–60 h/week or less than 40 h/week. 

Poisson regression analyses were performed to explore whether working status itself was associated with ULBs (Table 3). After adjusting for age, sex, educational level, and household income, workers were more likely to be current smokers, heavy drinkers, and physically inactive than nonworkers (adjusted odds ratio [OR] = 1.48 [95% confidence interval (CI) = 1.41–1.56], 1.79 [95% CI = 1.68–1.90], and 1.07 [95% CI = 1.04–1.11], respectively).

**Figure 2:**
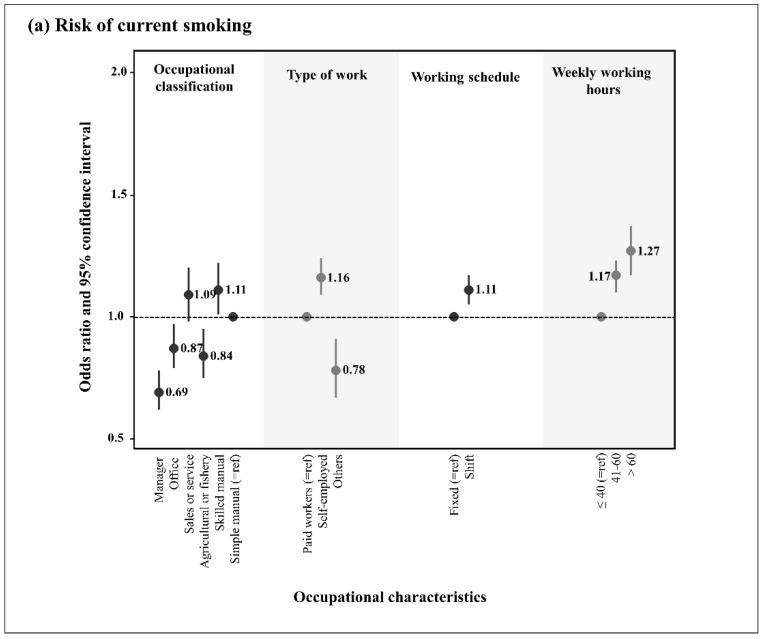

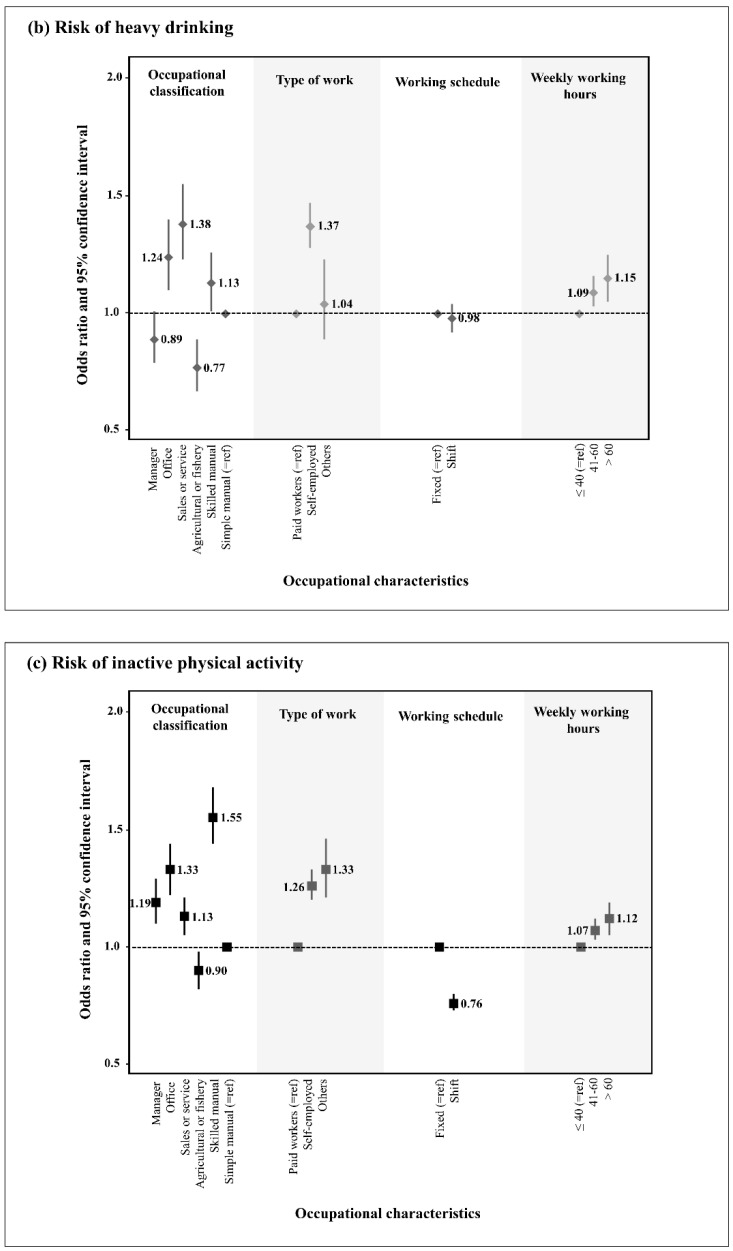
Results from the Poisson regression analyses for unhealthy lifestyle behaviors according to occupational characteristics among workers.

The association between each ULB and occupational characteristics based on occupational classification, employment status, work schedule, and weekly working hours is shown in Figure 2. Considering simple manual workers as the reference group, only skilled manual workers showed significantly increased odds of all three ULBs. In contrast, agricultural or fishery workers showed significantly reduced odds for all three ULBs. Sales and service workers showed higher odds of all three ULBs, although the difference in current smoking did not reach statistical significance. Office workers showed reduced odds of current smoking, but increased odds of heavy drinking and physical inactivity. Managers had reduced odds of current smoking and increased odds of physical inactivity. However, the odds of heavy drinking did not reach statistical significance for the managers. Using paid workers as the reference group, self-employed workers showed a higher risk of current smoking, heavy drinking, and physical inactivity. In terms of work schedule, shift workers were more likely to be current smokers and less likely to be physically inactive than day workers. Regarding weekly working hours, workers working over 60 h/week and 41–60 h/week had greater odds of current smoking, heavy drinking, and physical inactivity than those working less than 40 h/week.

Further analyses were conducted to investigate the association between the status of ULB and occupational classification and the type of hiring (direct contracted and dispatched workers). Results can find in the supplementary tables. 

## 4. Discussion

Our nationwide cross-sectional study showed that workers were more likely than nonworkers to engage in ULBs such as current smoking, heavy drinking, and physical inactivity. Among workers, the risk of engaging in ULBs differed significantly according to occupational characteristics, such as occupational classification, employment status, working schedule, and weekly working hours. 

In assessments based on sociodemographic factors, current smoking and heavy drinking were more prevalent in males, younger adults, and those with higher incomes. These findings were not limited to workers, but to all participants. Sex-related differences in current smoking and heavy drinking have been well documented in previous studies, with a higher prevalence of smoking and heavy drinking reported in males across different societies and cultures [23,24,25]. One literature review hypothesized that females are more likely to be responsive to health concerns and have a higher expectation of self-control than males [26], while traditional social and gender norms for females may also result in sex-related differences in smoking and alcohol consumption [27,28]. 

Our data also showed that physical inactivity was more prevalent among females than among males. Greater PA in males has previously been reported [29], and may be related to traditional gender roles, wherein females are expected to serve as caregivers at home and have less time to participate in physical activities [30,31]. However, sex differences in PA disappeared when the subjects were limited to the working population. Higher socioeconomic status, often measured as a combination of education, income, and occupation, has been positively associated with PA [32,33]. In addition, some evidence shows that working women are more physically active than nonworking women [34,35]. This could be because working women have better financial conditions and more resources, allowing them to engage in more PA. Another explanation may be that working women no longer perceive their gender as a constraint. Consequently, they are less likely to be constrained by familial or social obligations and are more likely to be involved in decision-making regarding their leisure time activities [36,37]. 

Our data on the age-related prevalence of current smoking and heavy drinking were similar to those previously reported. In general, the prevalence of current smoking and heavy drinking is considerably higher in young adulthood and decreases in elderly people [38,39]. Older individuals are more likely to participate in healthy lifestyle behaviors because of their higher probability of morbidity and mortality [40]. 

Our data showed that middle-aged (41–60 years) and older (>60 years) groups were more physically inactive than the younger age group (19–40 years). A reduction in PA with age is one of the most consistent observations in behavioral epidemiology [41,42]. Specifically, some cross-sectional studies have shown a more prominent decrease in PA after 45–50 years of age [43,44], which may be a consequence of aging-induced structural and functional alterations in the cardiopulmonary and musculoskeletal systems [45].

The association between socioeconomic status and ULBs has previously been reported previously, and a lower socioeconomic status has been suggested to increase the likelihood of ULBs, such as current smoking [46,47], heavy drinking [48], and physical inactivity [49,50]. However, our findings were inconsistent with these results because the prevalence of current smoking and heavy drinking was higher in the third and fourth quartiles of household income in this study. Only physical inactivity was more common in the lowest income group. These inconsistencies may be explained by the different sociocultural conditions across countries and variations in the assessment of socioeconomic status and behavioral patterns. However, one interesting finding in our study was that, in analyses of the working population, physical inactivity was more prevalent in the fourth quartile of household income. This can be explained by the strong influence of occupation on daily PA, as most adults spend most of their waking hours at work. Moreover, high-income individuals are more likely to hold sedentary jobs, thus minimizing their time to engage in PA [51,52]. 

After adjusting for sociodemographic variables (age, sex, educational level, and household income), workers were more likely to engage in the three ULBs than were nonworkers. Thus, work-related conditions may promote ULBs among workers. Stressful working conditions are known to result in ULBs. One way to explain the influence of stress on ULBs is that it challenges the body’s ability to maintain physiological and psychological stability. Alcohol is often consumed for relaxation in response to stress [53], and self-control is a limited resource; therefore, greater levels of stress can deplete self-control and promote ULBs [54]. Nicotine induces the hypothalamic–pituitary–adrenal axis, activates the autonomic nervous system functioning between stress and smoking [55], and induces dopaminergic neurotransmission in the mesolimbic regions of the brain to reinforce drug cravings and abuse [56]. Higher stress was also significantly associated with lower self-centered PA. Lack of time and fatigue, which are potential side effects of stress, have been reported as the main causes of physical inactivity [57]. In a study on sedentary behavior and perceived stress by occupation, white-collar workers who had more sedentary behavior were associated with higher levels of perceived stress [58]. 

The intensity of job stress varies depending on occupational characteristics, such as occupational classification, employment status, shift work, and working hours. In occupational classification, the skilled manual group had higher ORs for all items of the ULB. The office and sales and service groups were associated with a higher risk of engaging in heavy drinking and inactive PA. The manager group also had a higher ORs for inactive PA. In workplaces with unsupported bosses, female managers and experts may be more likely to engage in unhealthy coping behaviors to manage stress [59]. There have been studies on high levels of job stress in sales and service groups [60,61,62]. 

The higher ORs of all ULB items among the skilled manual group may be explained by the fact that the skilled manual group is required to have a higher level of job demands than the simple manual group. In addition, the fact that the office group had higher ORs for all ULB items than the manager group may be associated with lower job control and satisfaction in the office group than in the manager group, which consists of the same white-collar worker. Therefore, our results suggest that different ORs of ULB in different occupations are related to job stress. 

Shift workers experience continuous stress driven by the need to adjust rapidly to variable work schedules [63]. Shift workers show higher job stress, psychological effects, and lower job satisfaction than daytime workers, and ULBs are more prevalent among shift workers than among daytime workers [64]. In this study, shift workers showed a higher risk of current smoking than daytime workers; however, they did not show a significantly higher risk of heavy drinking, and even showed more PA. Consequently, our analyses of the relationship between shift work and ULBs were limited, and more sophisticated analyses based on the characteristics of shift work, such as shift work type, hours, and direction, are needed in this regard. 

In this study, long working hours were associated with current smoking, heavy drinking, and physical inactivity. Considering the group with fewer than 40 working hours as the reference, the risks of current smoking, heavy drinking, and physical inactivity increased with an increase in working hours. This result was consistent with other studies in which long working hours were significantly related to psychosocial stress responses among Korean [65,66] and Chinese workers [67].

The major strength of this study is that it is the first study, to the best of our knowledge, to compare the risk of ULBs between workers and nonworkers, and according to various occupational characteristics among workers. Moreover, the study participants were selected from the nationwide well-characterized KNHANES dataset, and the sample size was sufficiently large to reliably represent the Korean population. Further longitudinal studies are needed to verify the causality of job stress and ULBs, and this study could serve as the basis for future studies. 

### Limitation 

This was a cross-sectional survey; thus, it showed a limited ability to infer causality between job stress and ULBs. Second, this study was based on questionnaire assessments, and as such was not free from response bias, and the stress level was not measured quantitatively. In addition, it was not possible to analyze the effect of job stress on unhealthy behaviors in specific occupations. Moreover, our results were limited because we did not fully adjust for confounders, including personal traits or environmental factors affecting stress. In addition, mediators such as depression and anxiety that lead to ULBs should be considered. 

## 5. Conclusions

In conclusion, this study aimed to demonstrate the differences in ULB between workers and nonworkers. The findings suggest that the risk of all ULBs was significantly higher in the working population. In addition, the results highlighted statistically significant differences in the risk of ULBs according to occupational classification, employment status, working schedule, and weekly working hours in the working population. These findings will be of value in ULB and workers’ health-related research. Further studies regarding the role of occupational characteristics in ULBs are warranted.

## Figures and Tables

**Table 1:** Descriptive characteristics of the study participants.

	Overall Study Population	Current Smoking, n (%)		*p*-Value	Heavy Drinking, n (%)		*p*-Value	Physical Activity, n (%)		*p*-Value
		No	Yes		No	Yes		Active	Inactive	
Total participants	72,665	58,548	14,117		64,704	7961		29,183	43,482	
Sex				<0.0001			<0.0001			<0.0001
Male	31,103	19,140 (61.5)	11,963 (38.5)		25,019 (80.4)	6084 (19.6)		13,213 (42.5)	17,890 (57.5)	
Female	41,562	39,408 (94.8)	2154 (5.2)		39,685 (95.5)	1877 (4.5)		15,970 (38.4)	25,592 (61.6)	
Age (years)				<0.0001			<0.0001			<0.0001
19–40	23,185	17,407 (75.1)	5778 (24.9)		19,912 (85.9)	3273 (14.1)		9818 (42.3)	13,367 (57.7)	
41–60	26,954	21,415 (79.4)	5539 (20.6)		23,388 (86.8)	3566 (13.2)		10,376 (38.5)	16,578 (61.5)	
>60	22,526	19,726 (87.6)	2800 (12.4)		21,404 (95.0)	1122 (5.0)		8989 (39.9)	13,537 (60.1)	
Education				<0.0001			<0.0001			<0.0001
Middle school or less	25,176	21,410 (85.0)	3766 (15.0)		23,312 (92.6)	1864 (7.4)		9439 (37.5)	15,737 (62.5)	
High school	24,314	18,547 (76.3)	5767 (23.7)		20,993 (86.3)	3321 (13.7)		10,399 (42.8)	13,915 (57.2)	
College or more	23,175	18,591 (80.2)	4584 (19.8)		20,399 (88.0)	2776 (12.0)		9345 (40.3)	13,830 (59.7)	
Household income				<0.0001			<0.0001			<0.0001
First quartile	14,289	11,765 (82.3)	2524 (17.7)		13,257 (92.8)	1032 (7.2)		5493 (38.4)	8796 (61.6)	
Second quartile	18,267	14,537 (79.6)	3730 (20.4)		16,289 (89.2)	1978 (10.8)		7375 (40.4)	10,892 (59.6)	
Third quartile	19,643	15,528 (79.1)	4115 (20.9)		17,241 (87.8)	2402 (12.2)		7916 (40.3)	11,727 (59.7)	
Fourth quartile	20,466	16,718 (81.7)	3748 (18.3)		17,917 (87.5)	2549 (12.5)		8399 (41.1)	12,067 (58.9)	
Working status				<0.0001			<0.0001			<0.0001
Workers	43,161	32,495 (75.3)	10,666 (24.7)		36,869 (85.4)	6296 (14.6)		17,773 (41.2)	25,388 (58.8)	
Nonworkers	29,504	26,053 (88.3)	3451 (11.7)		27,835 (94.3)	1669 (5.7)		11,410 (38.7)	18,094 (61.3)	

**Table 2:** Descriptive characteristics of study participants according to the occupational characteristics of the working population.

	Overall Study Population	Current Smoking, n (%)		*p*-Value	Heavy Drinking, n (%)		*p*-Value	Physical Activity, n (%)		*p*-Value
		No	Yes		No	Yes		Active	Inactive	
Total workers	42,870	32,300	10,570		36,632	6238		17,274	25,596	
Sex				<0.0001			<0.0001			0.3772
Male	22,602	13,172 (58.3)	9430 (41.7)		17,562 (77.7)	5040 (22.3)		9152 (40.5)	13,450 (59.5)	
Female	20,268	19,128 (94.4)	1140 (5.6)		19,070 (94.1)	1198 (5.9)		8122 (40.1)	12,146 (59.9)	
Age (years)				<0.0001			<0.0001			<0.0001
19–40	14,706	10,172 (69.2)	4534 (30.8)		12,145 (82.6)	2561 (17.4)		6338 (43.1)	8368 (56.9)	
41–60	19,545	14,873 (76.1)	4672 (23.9)		16,469 (84.3)	3076 (15.7)		7414 (37.8)	12,131 (62.1)	
>60	8619	7255 (84.2)	1364 (15.8)		8018 (93.0)	601 (7.0)		3522 (40.9)	5097 (59.1)	
Education				<0.0001			<0.0001			0.1151
Middle school or less	12,246	9924 (81.0)	2322 (19.0)		10,952 (89.4)	1294 (10.6)		4754 (38.8)	7492 (61.2)	
High school	14,532	10,214 (70.3)	4318 (29.7)		12,007 (82.6)	2525 (17.4)		6087 (41.9)	8445 (58.1)	
College or more	16,092	12,162 (75.6)	3930 (24.4)		13,673 (85.0)	2419 (15.0)		6433 (40.0)	9659 (60.0)	
Household income				<0.0001			<0.0001			0.0093
First quartile	5459	4309 (78.9)	1150 (21.1)		4925 (90.2)	534 (9.8)		2298 (42.1)	3161 (57.9)	
Second quartile	10,556	7802 (73.9)	2754 (26.1)		9055 (85.8)	1501 (14.2)		4281 (40.6)	6275 (59.4)	
Third quartile	12,716	9291 (73.1)	3425 (26.9)		10,735 (84.4)	1981 (15.6)		5045 (39.7)	7671 (60.3)	
Fourth quartile	14,1390	10,898 (77.1)	3241 (22.9)		11,917 (84.3)	2222 (15.7)		5650 (40.0)	8489 (60.0)	
Occupational classification				<0.0001			<0.0001			0.6472
Manager	9295	7363 (79.2)	1932 (20.8)		8091 (87.1)	1204 (12.9)		3841 (41.3)	5454 (58.7)	
Office	6539	4963 (75.9)	1576 (24.1)		5469 (83.4)	1070 (16.4)		2570 (39.9)	3969 (60.7)	
Sales or service	9075	6985 (77.0)	2090 (23.0)		7621 (84.0)	1454 (16.0)		3713 (40.9)	5362 (59.1)	
Agricultural or fishery	4671	3729 (79.8)	942 (20.2)		4210 (90.1)	461 (9.9)		1924 (41.2)	2747 (58.8)	
Skilled manual	6954	4110 (59.1)	2844 (40.9)		5513 (79.3)	1441 (20.7)		2411 (34.7)	4543 (65.3)	
Simple manual	6336	5150 (81.3)	1186 (18.7)		5728 (90.4)	608 (9.6)		2815 (44.4)	3521 (55.6)	
Employment status				<0.0001			<0.0001			<0.0001
Paid workers	27,644	20,845 (75.4)	6799 (24.6)		23,743 (85.9)	3901 (14.1)		11,622 (42.0)	16,022 (58.0)	
Self-employed	12,450	8930 (71.7)	3520 (28.3)		10,311 (82.8)	2139 (17.2)		4631 (37.2)	7819 (62.8)	
Others	2776	2525 (91.0)	251 (9.0)		2578 (92.9)	198 (7.1)		1021 (36.8)	1755 (63.2)	
Working schedule				<0.0001			0.0021			<0.0001
Daytime fixed	28,119	21,595 (76.8)	6524 (23.2)		24,134 (85.8)	3985 (14.2)		10,661 (37.9)	17,458 (62.1)	
Shift	14,751	10,705 (72.6)	4046 (27.4)		12,498 (84.7)	2253 (15.3)		6613 (44.8)	8138 (55.2)	
Weekly working hours				<0.0001			<0.0001			<0.0001
≤40	21,280	17,222 (80.9)	4058 (19.1)		18,758 (88.1)	2522 (11.9)		8787 (41.3)	12,493 (58.7)	
41–60	16,201	11,400 (70.4)	4801 (29.6)		13,456 (83.1)	2745 (16.9)		6412 (39.6)	9789 (60.4)	
>60	5389	3678 (68.3)	1711 (31.7)		4418 (82.0)	971 (18.0)		2075 (38.5)	3314 (61.5)	

**Table 3:** Results from the Poisson regression analyses for unhealthy lifestyle behaviors according to working status.

	Working Status, Odds Ratio (95% Confidence Intervals)	
	Nonworkers	Workers
Current smoking	Reference	1.33 (1.28–1.39)
Heavy drinking	Reference	1.66 (1.57–1.76)
Physical inactivity	Reference	1.03 (1.01–1.05)

All results were adjusted for age, sex, educational level, and household income.

## Data Availability

All data from the KNHANES are accessible at: https://knhanes.kdca.go.kr/knhanes/eng/index.do (accessed on 15 July 2022).

## Supplementary Materials

The following supporting information can be downloaded at: https://www.mdpi.com/article/10.3390/ijerph192013021/s1.
