# Supplementary material for: Status of Workers’ Health Behavior and the Association between Occupational Characteristics and Health Behavior
Source: Int J Environ Res Public Health. 2022 Oct 11;19(20):13021. doi: 10.3390/ijerph192013021 (PMC9602100; doi:10.3390/ijerph192013021)
Supplement: Supplementary file 1 [file ijerph-19-13021-s001.zip › ijerph-1896756-supplementary.pdf]

Supplementary Table S1. Status and risk of unhealthy lifestyle behaviors among 'Manager' of occupational classification.

Occupational classification	Overall	Current smoking, n (%)		P-value	Heavy drinking, n (%)		P-value	Physical activity, n (%)		P-value
		No	Yes		No	Yes		Active	Inactive	
Manager	9,295	7,363 (79.2)	1,932 (20.8)		8,091 (87.0)	1,204 (13.0)		3,841 (41.3)	5,454 (58.7)	
Sex				<.0001			<.0001			0.0168
Male	4,906	3,142 (64.0)	1,764 (36.0)		3,905 (79.6)	1,001 (20.4)		2,084 (42.5)	2,822 (57.5)	
Female	4,389	4,221 (96.2)	168 (3.8)		4,186 (95.4)	203 (4.6)		1,757 (40.0)	2,632 (60.0)	
Age (years)				0.1716			0.5891			0.1220
19–40	4,880	3,868 (79.3)	1,012 (20.7)		4,280 (87.7)	600 (12.3)		2,101 (43.1)	2,779 (56.9)	
41–60	3,878	3,035 (78.3)	843 (21.7)		3,322 (85.7)	556 (14.3)		1,478 (38.1)	2,400 (61.9)	
>60	537	460 (85.7)	77 (14.3)		489 (91.1)	48 (8.9)		262 (48.8)	275 (51.2)	
Education				<.0001			<.0001			<.0001
Middle school or less	178	132 (74.2)	46 (25.8)		156 (87.6)	22 (12.4)		91 (51.1)	87 (48.9)	
High school	1,675	1,210 (72.2)	465 (27.8)		1,378 (82.3)	297 (17.7)		773 (46.1)	902 (53.9)	
College or more	7,442	6,021 (80.9)	1,421 (19.1)		6,557 (88.1)	885 (11.9)		2,977 (40.0)	4,465 (60.0)	
Household income				0.0094			0.5571			0.0040
First quartile	341	269 (78.9)	72 (21.1)		308 (90.3)	33 (9.7)		162 (47.5)	179 (52.5)	
Second quartile	1,413	1,111 (78.6)	302 (21.4)		1,241 (87.8)	172 (12.2)		627 (44.4)	786 (55.6)	
Third quartile	2,792	2,145 (76.8)	647 (23.2)		2,391 (85.6)	401 (14.4)		1,121 (40.2)	1,671 (59.8)	
Fourth quartile	4,749	3,838 (80.8)	911 (19.2)		4,151 (87.4)	598 (12.6)		1,931 (40.7)	2,818 (59.3)	
Employment status				<.0001			<.0001			0.0513
Paid workers	6,778	5,477 (80.8)	1,301 (19.2)		5,987 (88.3)	791 (11.7)		2,839 (41.9)	3,939 (58.1)	
Self-employed	2,460	1,840 (74.8)	620 (25.2)		2,057 (83.6)	403 (16.4)		983 (40.0)	1,477 (60.0)	
Others	57	46 (80.7)	11 (19.3)		47 (82.5)	10 (17.5)		19 (33.3)	38 (66.7)	
Working schedule				0.0594			0.9689			0.3799
Daytime fixed	6,281	5,010 (79.8)	1,271 (20.2)		5,468 (87.1)	813 (12.9)		2,576 (41.0)	3,705 (59.0)	
Shift	3,014	2,353 (78.1)	661 (21.9)		2,623 (87.0)	391 (13.0)		1,265 (42.0)	1,749 (58.0)	
Weekly working hours				<.0001			<.0001			0.4274
≤40	5,145	4,319 (83.9)	826 (16.1)		4,573 (88.9)	572 (11.1)		2,150 (41.8)	2,995 (58.2)	
41–60	3,559	2,670 (75.0)	889 (25.0)		3,045 (85.6)	514 (14.4)		1,446 (40.6)	2,113 (59.4)	
>60	591	374 (63.3)	217 (36.7)		473 (80.0)	118 (20.0)		245 (41.5)	346 (58.5)	

Supplementary Table S2. Status and risk of unhealthy lifestyle behaviors among 'Office' of occupational classification.

Occupational classification	Overall	Current smoking, n (%)		P-value	Heavy drinking, n (%)		P-value	Physical activity, n (%)		P-value
		No	Yes		No	Yes		Active	Inactive	
Office	6.539	4,963 (75.9)	1,576 (24.1)		5,469 (83.6)	1,070 (16.4)		2,570 (39.3)	3,969 (60.7)	
Sex				<.0001			<.0001			0.0353
Male	3,431	2,018 (58.8)	1,413 (41.2)		2,567 (74.8)	864 (25.2)		1,390 (40.5)	2,041 (59.5)	
Female	3,108	2,945 (94.8)	163 (5.2)		2,902 (93.4)	206 (6.6)		1,180 (38.0)	1,928 (62.0)	
Age (years)				0.0004			0.6148			0.4808
19–40	3,538	2,637 (74.5)	901 (25.5)		2,960 (83.7)	578 (16.3)		1,430 (40.4)	2,108 (59.6)	
41–60	2,722	2,092 (76.9)	630 (23.1)		2,266 (83.2)	456 (16.8)		1,007 (37.0)	1,715 (63.0)	
>60	279	234 (83.9)	45 (16.1)		243 (87.1)	36 (12.9)		133 (47.7)	146 (52.3)	
Education				0.6206			0.0888			0.7743
Middle school or less	180	134 (74.4)	46 (25.6)		157 (87.2)	23 (12.8)		62 (34.4)	118 (65.6)	
High school	2,093	1,603 (76.6)	490 (23.4)		1,765 (84.3)	328 (15.7)		834 (39.8)	1,259 (60.2)	
College or more	4,266	3,226 (75.6)	1,040 (24.4)		3,547 (83.1)	719 (16.9)		1,674 (39.2)	2,592 (60.8)	
Household income				<.0001			0.2646			0.0576
First quartile	195	147 (75.4)	48 (24.6)		167 (85.6)	28 (14.4)		92 (47.2)	103 (52.8)	
Second quartile	1,110	804 (72.4)	306 (27.6)		933 (84.1)	177 (15.9)		447 (40.3)	663 (59.7)	
Third quartile	2,157	1,579 (73.2)	578 (26.8))		1,811 (84.0)	346 (16.0)		843 (39.1)	1,314 (60.9)	
Fourth quartile	3,077	2,433 (79.1)	644 (20.9)		2,558 (83.1)	519 (16.9)		1,188 (38.6)	1,889 (61.4)	
Employment status				0.2157			0.5528			0.4526
Paid workers	5,905	4,498 (76.2)	1,407 (23.8)		4,953 (83.9)	952 (16.1)		2,328 (39.4)	3,577 (60.6)	
Self-employed	467	305 (65.3)	162 (34.7)		355 (76.0)	112 (24.0)		181 (38.8)	286 (61.2)	
Others	167	160 (95.8)	7 (4.2)		161 (96.4)	6 (3.6)		61 (36.5)	106 (63.5)	
Working schedule				<.0001			0.4327			0.8680
Daytime fixed	5,055	3,896 (77.1)	1,159 (22.9)		4,218 (83.4)	837 (16.6)		1,984 (39.2)	3,071 (60.8)	
Shift	1,484	1,067 (71.9)	417 (28.1)		1,251 (84.3)	233 (15.7)		586 (39.5)	898 (60.5)	
Weekly working hours				<.0001			<.0001			0.2655
≤40	3,612	2,925 (81.0)	687 (19.0)		3,100 (85.8)	512 (14.2)		1,445 (40.0)	2,167 (60.0)	
41–60	2,681	1,891 (70.5)	790 (29.5)		2,188 (81.6)	493 (18.4)		1,028 (38.3)	1,653 (61.7)	
>60	246	147 (59.8)	99 (40.2)		181 (73.6)	65 (26.4)		97 (39.4)	149 (60.6)	

Supplementary Table S3. Status and risk of unhealthy lifestyle behaviors among 'Sales or service' of occupational classification.

Occupational classification	Overall	Current smoking, n (%)		P-value	Heavy drinking, n (%)		P-value	Physical activity, n (%)		P-value
		No	Yes		No	Yes		Active	Inactive	
Sales or service	9,075	6,985 (77.0)	2,090 (23.0)		7,621 (84.0)	1,454 (16.0)		3,713 (40.9)	5,362 (59.1)	
Sex				<.0001			<.0001			0.9297
Male	3,368	1,756 (52.1)	1,612 (47.9)		2,465 (73.2)	903 (26.8)		1,380 (41.0)	1,988 (59.0)	
Female	5,707	5,229 (91.6)	478 (8.4)		5,156 (90.3)	551 (9.7)		2,333 (40.9)	3,374 (59.1)	
Age (years)				<.0001			<.0001			0.0009
19–40	3,162	2,005 (63.4)	1,157 (36.6)		2,445 (77.3)	717 (22.7)		1,408 (44.5)	1,754 (55.5)	
41–60	4,720	3,918 (83.0)	802 (17.0)		4,055 (85.9)	665 (14.1)		1,805 (38.2)	2,915 (61.8)	
>60	1,193	1,062 (89.0)	131 (11.0)		1,121 (94.0)	72 (6.0)		500 (41.9)	693 (58.1)	
Education				<.0001			<.0001			0.1669
Middle school or less	2,361	2,042 (86.5)	319 (13.5)		2,113 (89.5)	248 (10.5)		907 (38.4)	1,454 (61.6)	
High school	4,403	3,333 (75.7)	1,070 (24.3)		3,653 (83.0)	750 (17.0)		1,873 (42.5)	2,530 (57.5)	
College or more	2,311	1,610 (69.7)	701 (30.3)		1,855 (80.3)	456 (19.7)		933 (40.4)	1,378 (59.6)	
Household income				0.3399			0.0349			0.0079
First quartile	964	762 (79.0)	202 (21.0)		839 (87.0)	125 (13.0)		425 (44.1)	539 (55.9)	
Second quartile	2,463	1,892 (76.8)	571 (23.2)		2,074 (84.2)	389 (15.8)		1,050 (42.6)	1,413 (57.4)	
Third quartile	2,841	2,172 (76.5)	669 (23.5)		2,359 (83.0)	482 (17.0)		1,109 (39.0)	1,732 (61.0)	
Fourth quartile	2,807	2,159 (76.9)	648 (23.1)		2,349 (83.7)	458 (16.3)		1,129 (40.2)	1,678 (59.8)	
Employment status				0.0290			0.0116			<.0001
Paid workers	4,789	3,694 (77.1)	1,095 (22.9)		4,094 (85.5)	695 (14.5)		2,192 (45.8)	2,597 (54.2)	
Self-employed	3,636	2,728 (75.0)	908 (25.0)		2,964 (81.5)	672 (18.5)		1,292 (35.5)	2,344 (64.5)	
Others	650	563 (86.6)	87 (13.4)		563 (86.6)	87 (13.4)		229 (35.2)	421 (64.80	
Working schedule				<.0001			<.0001			<.0001
Daytime fixed	5,338	4,265 (79.9)	1,073 (20.1)		4,571 (85.6)	767 (14.4)		2,059 (38.6)	3,279 (61.4)	
Shift	3,737	2,720 (72.8)	1,017 (27.2)		3,050 (81.6)	687 (18.4)		1,654 (44.3)	2,083 (55.7)	
Weekly working hours				<.0001			<.0001			<.0001
≤40	4,009	3,314 (82.7)	695 (17.3)		3,508 (87.5)	501 (12.5)		1,802 (44.9)	2,207 (55.1)	
41–60	3,090	2,226 (72.0)	864 (28.0)		2,493 (80.7)	597 (19.3)		1,191 (38.5)	1,899 (61.5)	
>60	1,976	1,445 (73.1)	531 (26.9)		1,620 (82.0)	356 (18.0)		720 (36.4)	1,256 (63.6)	

Supplementary Table S4. Status and risk of unhealthy lifestyle behaviors among 'Agricultural or fishery' of occupational classification.

Occupational classification	Overall	Current smoking, n (%)		P-value	Heavy drinking, n (%)		P-value	Physical activity, n (%)		P-value
		No	Yes		No	Yes		Active	Inactive	
Agricultural or fishery	4,671	3,729 (79.8)	942 (20.2)		4,210 (90.1)	461 (9.9)		1,924 (41.2)	2,747 (58.8)	
Sex				<.0001			<.0001			<.0001
Male	2,578	1,674 (64.9)	904 (35.1)		2,144 (83.2)	434 (16.8)		1,149 (44.6)	1,429 (55.4)	
Female	2,093	2,055 (98.2)	38 (1.8)		2,066 (98.7)	27 (1.3)		775 (37.0)	1,318 (63.0)	
Age (years)				<.0001			<.0001			0.0205
19–40	173	116 (67.1)	57 (32.9)		142 (82.1)	31 (17.9)		85 (49.1)	88 (50.9)	
41–60	1,652	1,239 (75.0)	413 (25.0)		1,397 (84.6)	255 (15.4)		697 (42.2)	955 (57.8)	
>60	2,846	2,374 (83.4)	472 (16.6)		2,671 (93.9)	175 (6.1)		1,142 (40.1)	1,704 (59.9)	
Education				<.0001			<.0001			0.0127
Middle school or less	3,624	3,015 (83.2)	609 (16.8)		3,353 (92.5)	271 (7.5)		1,461 (40.3)	2,163 (59.7)	
High school	792	545 (68.8)	247 (31.2)		650 (82.1)	142 (17.9)		343 (43.3)	449 (56.7)	
College or more	255	169 (66.3)	86 (33.7)		207 (81.2)	48 (18.8)		120 (47.1)	135 (52.9)	
Household income				0.0191			<.0001			0.2100
First quartile	1,675	1,358 (81.1)	317 (18.9)		1,562 (93.3)	113 (6.7)		683 (40.8)	992 (59.2)	
Second quartile	1,412	1,129 (80.0)	283 (20.0)		1,261 (89.3)	151 (10.7)		551 (39.0)	861 (61.0)	
Third quartile	862	691 (80.2)	171 (19.8)		763 (88.5)	99 (11.5)		392 (45.5)	470 (54.5)	
Fourth quartile	722	551 (76.3)	171 (23.7)		624 (86.4)	98 (13.6)		298 (41.3)	424 (58.7)	
Employment status				<.0001			<.0001			<.0001
Paid workers	135	93 (68.9)	42 (31.1)		108 (80.0)	27 (20.0)		77 (57.0)	58 (43.0)	
Self-employed	3,159	2,320 (73.4)	839 (26.6)		2,759 (87.3)	400 (12.7)		1,342 (42.5)	1,817 (57.5)	
Others	1,377	1,316 (95.6)	61 (4.4)		1,343 (97.5)	34 (2.5)		505 (36.7)	872 (63.3)	
Working schedule				0.5121			0.8681			<.0001
Daytime fixed	3,003	2,406 (80.1)	597 (19.9)		2,705 (90.1)	298 (9.9)		1,030 (34.3)	1,973 (65.7)	
Shift	1,668	1,323 (79.3)	345 (20.7)		1,505 (90.2)	163 (9.8)		894 (53.6)	774 (46.4)	
Weekly working hours				<.0001			0.0318			<.0001
≤40	2,301	1,889 (82.1)	412 (17.9)		2,104 (91.4)	197 (8.6)		827 (35.9)	1,474 (64.1)	
41–60	1,649	1,298 (78.7)	351 (21.3)		1,458 (88.4)	191 (11.6)		745 (45.2)	904 (54.8)	
>60	721	542 (75.2)	179 (24.8)		648 (89.9)	73 (10.1)		352 (48.8)	369 (51.2)	

Supplementary Table S5. Status and risk of unhealthy lifestyle behaviors among 'Skilled manual' of occupational classification.

Occupational classification	Overall	Current smoking, n (%)		P-value	Heavy drinking, n (%)		P-value	Physical activity, n (%)		P-value
		No	Yes		No	Yes		Active	Inactive	
Skilled manual	6,954	4,110 (59.1)	2,844 (40.9)		5,513 (79.3)	1,441 (20.7)		2,411 (34.7)	4,543 (65.3)	
Sex				<.0001			<.0001			0.0380
Male	5,858	3,086 (52.7)	2,772 (47.3)		4,472 (76.3)	1,386 (23.7)		2,001 (3.42)	3,857 (65.8)	
Female	1,096	1,024 (93.4)	72 (6.6)		1,041 (95.0)	55 (5.0)		410 (37.4)	686 (62.6)	
Age (years)				<.0001			<.0001			<.0001
19–40	1,999	923 (46.2)	1,076 (53.8)		1,529 (76.5)	470 (23.5)		831 (41.6)	1,168 (58.4)	
41–60	3,903	2,410 (61.7)	1,493 (38.3)		3,062 (78.5)	841 (21.5)		1,251 (32.1)	2,652 (67.9)	
>60	1,052	777 (73.9)	275 (26.1)		922 (87.6)	130 (12.4)		329 (31.3)	723 (68.7)	
Education				<.0001			0.5402			<.0001
Middle school or less	2,279	1,507 (66.1)	772 (33.9)		1,820 (79.9)	459 (20.1)		698 (30.6)	1,581 (69.4)	
High school	3,368	1,856 (55.1)	1,512 (44.9)		2,629 (78.1)	739 (21.9)		1,225 (36.4)	2,143 (63.6)	
College or more	1,307	747 (57.2)	560 (42.8)		1,064 (81.4)	243 (18.60		488 (37.3)	819 (62.7)	
Household income				<.0001			0.0086			0.8721
First quartile	532	322 (60.5)	210 (39.5)		434 (81.6)	98 (18.4)		207 (38.9)	325 (61.1)	
Second quartile	2,059	1,189 (57.7)	870 (42.3)		1,638 (79.6)	421 (20.4)		689 (33.5)	1,370 (66.5)	
Third quartile	2,524	1,461 (57.9)	1,063 (42.1)		2,034 (80.6)	490 (19.4)		863 (34.2)	1,661 (65.8)	
Fourth quartile	1,839	1,138 (61.9)	701 (38.1)		1,407 (76.5)	432 (23.5)		652 (35.5)	1,187 (64.5)	
Employment status				0.0877			0.2697			<.0001
Paid workers	4,617	2,652 (57.4)	1,965 (42.6)		3,685 (79.8)	932 (20.2)		1,746 (37.8)	2,871 (62.2)	
Self-employed	2,168	1,325 (61.1)	843 (38.9)		1,689 (77.9)	479 (22.1)		599 (27.6)	1,569 (72.4)	
Others	169	133 (78.7)	36 (21.3)		139 (82.2)	30 (17.8)		66 (39.1)	103 (60.9)	
Working schedule				<.0001			0.8662			<.0001
Daytime fixed	4,499	2,737 (60.8)	1,762 (39.2)		3,564 (79.2)	935 (20.8)		1,397 (31.1)	3,102 (68.9)	
Shift	2,455	1,373 (55.9)	1,082 (44.1)		1,949 (79.4)	506 (20.6)		1,014 (41.3)	1,441 (58.7)	
Weekly working hours				0.0001			0.0279			0.0253
≤40	2,340	1,464 (62.6)	876 (37.4)		1,883 (80.5)	457 (19.5)		836 (35.7)	1,504 (64.3)	
41–60	3,549	2,041 (57.5)	1,508 (42.5)		2,808 (79.1)	741 (20.9)		1,241 (35.0)	2,308 (65.0)	
>60	1,065	605 (56.8)	460 (43.2)		822 (77.2)	243 (22.8)		334 (31.4)	731 (68.6)	

Supplementary Table S6. Status and risk of unhealthy lifestyle behaviors among 'Simple manual' of occupational classification.

Occupational classification	Overall	Current smoking, n (%)		P-value	Heavy drinking, n (%)		P-value	Physical activity, n (%)		P-value
		No	Yes		No	Yes		Active	Inactive	
Simple manual	6,336	5,150 (81.3)	1,186 (18.7)		5,728 (90.4)	608 (9.6)		2,815 (44.4)	3,521 (55.6)	
Sex				<.0001			<.0001			0.0046
Male	2,461	1,496 (60.8)	965 (39.2)		2,009 (81.6)	452 (18.4)		1,148 (46.6)	1,313 (53.4)	
Female	3,875	3,654 (94.3)	221 (5.7)		3,719 (96.0)	156 (4.0)		1,667 (43.0)	2,208 (57.0)	
Age (years)				<.0001			<.0001			0.0001
19–40	954	623 (65.3)	331 (34.7)		789 (82.7)	165 (17.3)		483 (50.6)	471 (49.4)	
41–60	2,670	2,179 (81.6)	491 (18.4)		2,367 (88.7)	303 (11.3)		1,176 (44.0)	1,494 (56.0)	
>60	2,712	2,348 (86.6)	364 (13.4)		2,572 (94.8)	140 (5.2)		1,156 (42.6)	1,556 (57.4)	
Education				<.0001			<.0001			0.0004
Middle school or less	3,624	3,094 (85.4)	530 (14.6)		3,353 (92.5)	271 (7.5)		1,535 (42.4)	2,089 (57.6)	
High school	2,201	1,667 (75.7)	534 (24.3)		1,932 (87.8)	269 (12.2)		1,039 (47.2)	1,162 (52.8)	
College or more	511	389 (76.1)	122 (23.9)		443 (86.7)	68 (13.3)		241 (47.2)	270 (52.8)	
Household income				0.6615			<.0001			0.0003
First quartile	1,752	1,451 (82.8)	301 (17.2)		1,615 (92.2)	137 (7.8)		729 (41.6)	1,023 (58.4)	
Second quartile	2,099	1,677 (79.9)	422 (20.1)		1,908 (90.9)	191 (9.1)		917 (43.7)	1,182 (56.3)	
Third quartile	1,540	1,243 (80.7)	297 (19.3)		1,377 (89.4)	163 (10.6)		717 (46.6)	823 (53.4)	
Fourth quartile	945	779 (82.4)	166 (17.6)		828 (87.6)	117 (12.4)		452 (47.8)	493 (52.2)	
Employment status				0.6261			0.2907			0.0174
Paid workers	5,420	4,431 (81.8)	989 (18.2)		4,916 (90.7)	504 (9.3)		2,440 (45.0)	2,980 (55.0)	
Self-employed	560	412 (73.6)	148 (26.4)		487 (87.0)	73 (13.0)		234 (41.8)	326 (58.2)	
Others	356	307 (86.2)	49 (13.8)		325 (91.3)	31 (8.7)		141 (39.6)	215 (60.4)	
Working schedule				<.0001			0.0001			<.0001
Daytime fixed	3,943	3,281 (83.2)	662 (16.8)		3,608 (91.5)	335 (8.5)		1,615 (41.0)	2,328 (59.0)	
Shift	2,393	1,869 (78.1)	524 (21.9)		2,120 (88.6)	273 (11.4)		1,200 (50.1)	1,193 (49.9)	
Weekly working hours				<.0001			<.0001			0.2788
≤40	3,873	3,311 (85.5)	562 (14.5)		3,590 (92.7)	283 (7.3)		1,727 (44.6)	2,146 (55.4)	
41–60	1,673	1,274 (76.2)	399 (23.8)		1,464 (87.5)	209 (12.5)		761 (45.5)	912 (54.5)	
>60	790	565 (71.5)	225 (28.5)		674 (85.3)	116 (14.7)		327 (41.4)	463 (58.6)	

Supplementary Table S7. Status and risk of unhealthy lifestyle behaviors according to type of hiring (direct contracted and dispatched workers).

	Overall	Current smoking, n (%)		P-value	Heavy drinking, n (%)		P-value	Physical activity, n (%)		P-value
		No	Yes		No	Yes		Active	Inactive	
Total subgroup subjects	24,995	18,792 (75.2)	6,203 (24.8)		21,384 (85.6)	3,611 (14.4)		10,333 (41.3)	14,662 (58.7)	
Direct contracted workers	19,957	14,970 (75.0)	4,987 (25.0)		17,039 (85.4)	2,918 (14.6)		8,152 (40.8)	11,805 (59.2)	
Sex				<.0001			<.0001			0.5934
Male	10,998	6,500 (59.1)	4,498 (40.9)		8,604 (78.2)	2,394 (21.8)		4,474 (40.7)	6,524 (59.3)	
Female	8,959	8,470 (94.5)	489 (5.5)		8,435 (94.1)	524 (5.9)		3,678 (41.1)	5,281 (58.9)	
Age (years)				<.0001			<.0001			0.7224
19–40	8,636	6,133 (71.0)	2,503 (29.0)		7,215 (83.5)	1,421 (16.5)		3,611 (41.8)	5,025 (58.2)	
41–60	8,990	6,912 (76.9)	2,078 (23.1)		7,679 (85.4)	1,311 (14.6)		3,522 (39.2)	5,468 (60.8)	
>60	2,331	1,925 (82.6)	406 (17.4)		2,145 (92.0)	186 (8.0)		1,019 (43.7)	1,312 (56.3)	
Education				0.0462			0.0493			0.3072
Middle school or less	2,795	2,194 (78.5)	601 (21.5)		2,481 (88.8)	314 (11.2)		1,094 (39.1)	1,701 (60.9)	
High school	6,697	4,741 (70.8)	1,956 (29.2)		5,599 (83.6)	1,098 (16.4)		2,780 (41.5)	3,917 (58.5)	
College or more	10,465	8,035 (76.8)	2,430 (23.2)		8,959 (85.6)	1,506 (14.4)		4,278 (40.9)	6,187 (59.1)	
Household income				<.0001			0.0037			0.1389
First quartile	1,333	999 (74.9)	334 (25.1)		1,183 (88.7)	150 (11.3)		592 (44.4)	741 (55.6)	
Second quartile	4,346	3,150 (72.5)	1,196 (27.5)		3,708 (85.3)	638 (14.7)		1,779 (40.9)	2,567 (59.1)	
Third quartile	6,475	4,737 (73.2)	1,738 (26.8)		5,534 (85.5)	941 (14.5)		2,591 (40.0)	3,884 (60.0)	
Fourth quartile	7,803	6,084 (78.0)	1,719 (22.0)		6,614 (84.8)	1,189 (15.2)		3,190 (40.9)	4,613 (59.1)	
Occupational classification				<.0001			0.2456			0.4894
Manager	5,598	4,491 (80.2)	1,107 (19.8)		4,940 (88.2)	658 (11.8)		2,343 (41.9)	3,255 (58.1)	
Office	4,646	3,548 (76.4)	1,098 (23.6)		3,875 (83.4)	771 (16.6)		1,819 (39.2)	2,827 (60.8)	
Sales or service	3,323	2,509 (75.5)	814 (24.5)		2,781 (83.7)	542 (16.3)		1,382 (41.6)	1,941 (58.4)	
Agricultural or fishery	457	320 (70.0)	137 (30.0)		386 (84.5)	71 (15.5)		206 (45.1)	251 (54.9)	
Skilled manual	3,257	1,930 (59.3)	1,327 (40.7)		2,626 (80.6)	631 (19.4)		1,165 (35.8)	2,092 (64.2)	
Simple manual	2,676	2,172 (81.2)	504 (18.8)		2,431 (90.8)	245 (9.2)		1,237 (46.2)	1,439 (53.8)	
Working schedule				<.0001			0.2219			<.0001
Daytime fixed	14,514	11,123 (76.6)	3,391 (23.4)		12,419 (85.6)	2,095 (14.4)		5,741 (39.6)	8,773 (60.4)	
Shift	5,443	3,847 (70.7)	1,596 (29.3)		4,620 (84.9)	823 (15.1)		2,411 (44.3)	3,032 (55.7)	
Weekly working hours				<.0001			<.0001			<.0001

≤40	10,161	8,266 (81.3)	1,895 (18.7)		8,950 (88.1)	1,211 (11.9)		4,341 (42.7)	5,820 (57.3)	
41–60	8,107	5,672 (70.0)	2,435 (30.0)		6,750 (83.3)	1,357 (16.7)		3,184 (39.3)	4,923 (60.7)	
>60	1,689	1,032 (61.1)	657 (38.9)		1,339 (79.3)	350 (20.7)		627 (37.1)	1,062 (62.9)	
Dispatched workers	5,038	3,822 (75.9)	1,216 (24.1)		4,345 (86.2)	693 (13.8)				
Sex				<.0001			<.0001			0.0047
Male	1,992	1,042 (52.3)	950 (47.7)		1,544 (77.5)	448 (22.5)		911 (45.7)	1,081 (54.3)	
Female	3,046	2,780 (91.3)	266 (8.7)		2,801 (92.0)	245 (8.0)		1,270 (41.7)	1,776 (58.3)	
Age (years)				<.0001			<.0001			<.0001
19–40	2,159	1,500 (69.5)	659 (30.5)		1,803 (83.5)	356 (16.5)		1,079 (50.0)	1,080 (50.0)	
41–60	1,826	1,417 (77.6)	409 (22.4)		1,553 (85.0)	273 (15.0)		693 (38.0)	1,133 (62.0)	
>60	1,053	905 (85.9)	148 (14.1)		989 (93.9)	64 (6.1)		409 (38.8)	644 (61.2)	
Education				0.8661			0.9761			0.0012
Middle school or less	1,858	1,464 (78.8)	394 (21.2)		1,618 (87.1)	240 (12.9)		708 (38.1)	1,150 (61.9)	
High school	2,191	1,557 (71.1)	634 (28.9)		1,859 (84.8)	332 (15.2)		1,058 (48.3)	1,133 (51.7)	
College or more	989	801 (81.0)	188 (19.0)		868 (87.8)	121 (12.2)		415 (42.0)	574 (58.0)	
Household income				0.6224			0.0409			0.1395
First quartile	1,043	818 (78.4)	225 (21.6)		924 (88.6)	119 (11.4)		442 (42.4)	601 (57.6)	
Second quartile	1,588	1,163 (73.2)	425 (26.8)		1,369 (86.2)	219 (13.8)		677 (42.6)	911 (57.4)	
Third quartile	1,391	1,043 (75.0)	348 (25.0)		1,179 (84.8)	212 (15.2)		598 (43.0)	793 (57.0)	
Fourth quartile	1,016	798 (78.5)	218 (21.5)		873 (85.9)	143 (14.1)		464 (45.7)	552 (54.3)	
Occupational classification				<.0001			0.8227			0.0002
Manager	655	551 (84.1)	104 (15.9)		581 (88.7)	74 (11.3)		292 (44.6)	363 (55.4)	
Office	408	337 (82.6)	7 (17.4)		365 (89.5)	43 (10.5)		189 (46.3)	219 (53.7)	
Sales or service	1,460	1,125 (77.1)	335 (22.9)		1,238 (84.8)	222 (15.2)		699 (47.9)	761 (52.1)	
Agricultural or fishery	145	105 (72.4)	40 (27.6)		118 (81.4)	27 (18.6)		57 (39.3)	88 (60.7)	
Skilled manual	806	444 (55.1)	362 (44.9)		630 (78.2)	176 (21.8)		298 (37.0)	508 (63.0)	
Simple manual	1,564	1,260 (80.6)	304 (19.4)		1,413 (90.3)	151 (9.7)		646 (41.3)	918 (58.7)	
Working schedule				0.0002			<.0001			<.0001
Daytime fixed	3,369	2,609 (77.4)	760 (22.6)		2,952 (87.6)	417 (12.4)		1,366 (40.5)	2,003 (59.5)	
Shift	1,669	1,213 (72.7)	456 (27.3)		1,393 (83.5)	276 (16.5)		815 (48.8)	854 (51.2)	
Weekly working hours				<.0001			<.0001			0.0784
≤40	3,280	2,651 (80.8)	629 (19.2)		2,891 (88.1)	389 (11.9)		1,437 (43.8)	1,843 (56.2)	
41–60	1,365	904 (66.2)	461 (33.8)		1,130 (82.8)	235 (17.2)		596 (43.7)	769 (56.3)	
>60	393	267 (67.9)	126 (32.1)		324 (82.4)	69 (17.6)		148 (37.7)	245 (62.3)	
